# Retroperitoneal Continuous Local Antibiotic Perfusion for Refractory Pyogenic Vertebral Osteomyelitis: A Case Report

**DOI:** 10.7759/cureus.50636

**Published:** 2023-12-16

**Authors:** Shuhei Ohyama, Masahiro Inoue, Noriyasu Toshi, Kohei Okuyama, Soichiro Tokeshi, Noritaka Suzuki, Yasuhiro Shiga, Kazuhide Inage, Eguchi Yawara, Sumihisa Orita, Seiji Ohtori

**Affiliations:** 1 Department of Orthopedic Surgery, Graduate School of Medicine, Chiba University, Chiba, JPN; 2 Department of Orthopedic Surgery, Center for Frontier Medical Engineering, Chiba University, Chiba, JPN

**Keywords:** continuous local antibiotic perfusion, epidural abscess, iliopsoas abscess, retroperitoneal space, pyogenic spondylitis

## Abstract

Pyogenic vertebral osteomyelitis (PVO) is a prevalent infection in the elderly, frequently complicated by iliopsoas and epidural abscesses. Traditional treatments are often ineffective for refractory cases. In this report, a 76-year-old man with PVO, iliopsoas, and epidural abscess was unresponsive to antibiotics, presenting with severe lower back pain and functional impairments. A two-stage surgical intervention was implemented: anterior debridement, autogenous bone graft fixation, and novel application of retroperitoneal continuous local antibiotic perfusion (CLAP), followed by posterior fixation. A contrast test verified correct CLAP perfusion into the iliopsoas abscess and intervertebral disc space. Substantial improvements were noted postoperatively, including a marked reduction in pain, inflammation, and the size of both abscesses. In conclusion, this case demonstrates the feasibility and effectiveness of retroperitoneal CLAP in treating refractory PVO, offering a potential innovative solution for cases resistant to conventional therapies.

## Introduction

Vertebral osteomyelitis (VO) is a spinal infection resulting in inflammation and destruction of the vertebral bodies and intervertebral discs [1-3]. VO can be caused by bacteria, fungi, or other organisms, with Staphylococcus aureus being the most common pathogen of pyogenic vertebral osteomyelitis (PVO), responsible for 55%-80% of cases [1-3]. This condition is increasingly prevalent in the elderly [4]. The proportion of patients aged 60 years or more among patients with PVO is 52% in Japan, and a similar trend has been reported in several studies [5-7]. Outcomes of treatment of PVO such as the incidence of paralysis and mortality within one year are worse in the elderly than in the younger adults [5,6]. Thus, the treatment of PVO in the elderly is an important issue. Traditional treatment options for PVO include antibiotics and surgical intervention, as well as debridement and instrumentation, which are effective in some cases and ineffective in others [8-10]. When combined with iliopsoas abscess and epidural abscess, treatment becomes complex and demands careful management. Patients should be carefully monitored for the appearance of neurologic symptoms and, in some cases, strict bed rest is required [11-14]. Additionally, the duration of antibiotic therapy, including oral antibiotics, may require 6-12 weeks [15]. These factors are serious conditions strongly associated with poor clinical outcomes and higher mortality in patients with PVO [16-18].

Continuous local antibiotic perfusion (CLAP) has been reported to have good results in the treatment of difficult-to-treat infections and instrumentation-related infections [19-21]. CLAP administers appropriate (high) concentrations of antibiotics locally and reduces the side effects of antibiotics because it applies continuous drainage via negative pressure [19-21]. The antibiotic selected is Gentamicin because it is a bactericidal antibiotic, concentration-dependent, and can be measured in blood levels [13-15]. The usefulness of CLAP is that it can eliminate antimicrobial-resistant bacteria and biofilm-associated bacteria, and control implant-associated infections while sparing the implants. In spine surgery, CLAP has been reported to have favorable outcomes for postoperative surgical site infections [20]. Therefore, CLAP has been proposed as a novel approach to treating difficult-to-treat infections. Despite this, there have been no documented instances of its application for cases of PVO accompanied by iliopsoas abscess and epidural abscess. Conventionally, CLAP has been utilized within compact, enclosed spaces, such as the medullary cavity or subcutaneous tissue, because it requires negative pressure for continuous perfusion [19-21]. This requirement is likely why its application in more expansive spaces like the retroperitoneal region remains unexplored. The present article presents a unique case of PVO with iliopsoas abscess and epidural abscess, successfully managed with a retroperitoneal CLAP procedure.

## Case presentation

A 76-year-old man presented severe back pain and fever. His medical history included immunoglobulin A (IgA) nephropathy, atrial fibrillation, diabetes mellitus, and an abdominal aortic aneurysm that had been previously stented. Laboratory parameters showed a high white blood cell count (10,900 per μl) and elevated C-reactive protein (CRP, 34.48 mg/dL). He had no other infection source or previous infections and was diagnosed with PVO based on his spinal MRI findings. He had been treated with transvenous antibiotics and bed rest. Initially, he was treated with cefazolin sodium (1.0 g every 12 hours) based on the susceptibility of Escherichia coli detected in his first blood culture. However, his laboratory parameters did not fully improve after eight weeks of initial treatment. Subsequently, he was treated with tazobactam/piperacillin (4.5 g every 8 hours) because his second blood culture identified extended-spectrum beta-lactamase-producing Escherichia coli. Despite the second treatment in four weeks, there was no improvement in his laboratory parameters and back pain. As a result, after three months of treatment with transvenous antibiotics and bed rest, he was transferred to our hospital for surgical treatment. Upon admission, he had difficulty walking and sitting due to back pain but exhibited no neurological deficits. Laboratory parameters showed decreased hemoglobin (8.0 g/dL) and elevated CRP (3.64 mg/dL), suggesting chronic inflammation (Table 1). One month after the second blood culture, his third blood culture was taken and was negative at the time of admission to our hospital.

**Table 1:** Laboratory data

Variable	Date on admission to our hospital	7 days after the second surgery	14 days after the second surgery	42 days after the second surgery
Hemoglobin (g/dl)	8.0	11.4	10.6	9.7
Hematocrit (%)	26.8	35.4	33.4	30.8
Red blood cell count (per μl)	2.85×10^6^	3.90×10^6^	3.54×10^6^	3.22×10^6^
White blood cell count (per μl)	6,600	6,000	4,300	3,800
White blood cell differential (%)				
Neutrophils	78.3	77.2	71.4	75.8
Lymphocytes	15.8	16.0	20.5	16.9
Monocytes	3.8	5.3	5.1	5.7
Eosinophils	1.8	1.3	2.5	1.3
Basophils	0.3	0.2	0.5	0.3
Platelet count (per μl)	24.3×10^4^	15.1×10^4^	16.5×10^4^	16.1×10^4^
Sodium (mmol/liter)	138	137	140	139
Potassium (mmol/liter)	3.6	2.6	2.6	3.6
Chloride (mmol/liter)	101	93	95	101
Urea nitrogen (mg/dl)	8	11	10	14
Creatinine (mg/dl)	0.77	0.64	0.65	0.91
Total protein (g/dl)	6.4	-	5.5	5.8
Albumin (g/dl)	2.3	2.4	2.2	2.5
Aspartate aminotransferase (IU/l)	11	13	12	18
Alanine aminotransferase (IU/l)	4	7	4	10
C-reactive protein (mg/dl)	3.64	1.99	0.82	0.12
Gentamicin (μg/ml)	-	0.9	0.9	-

**Figure 1:**
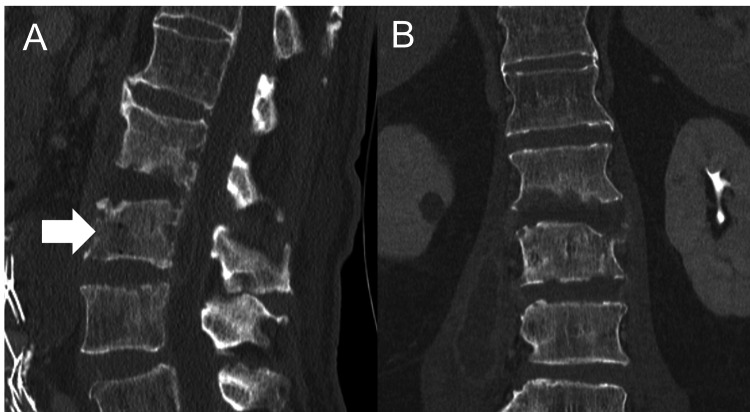
Preoperative computed tomography scan of the lumbar spine (A) sagittal image, (B) coronal image Computed tomography showing bone destruction at the L2 vertebral body and L3 vertebral body showing a gas-forming lesion (arrow).

**Figure 2:**
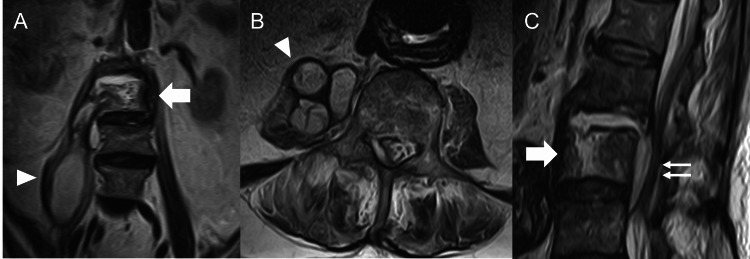
Preoperative magnetic resonance imaging of lumbar spine (A) coronal image, (B) axial image, (C) sagittal image T2-weighted magnetic resonance imaging showing multifocal right iliopsoas (arrowheads), epidural abscesses (double arrow), and erosion of the L3 vertebral body (arrows)

Radiological findings revealed diffuse idiopathic skeletal hyperostosis above the T12 vertebral body and bone destruction at the L2 inferior endplate and L3 superior endplate. Computed tomography showed bone destruction at the L2 vertebral body and L3 vertebral body showing a gas-forming infection (Figure 1). MRI showed high signal intensity at the L2-3 disc on T2-weighted images and a large multifocal cyst within the right iliopsoas muscle (Figures 2A, 2B). At the level of the L3 vertebral body, epidural fluid was shown on T2-weighted images (Figure 2C).

**Figure 3:**
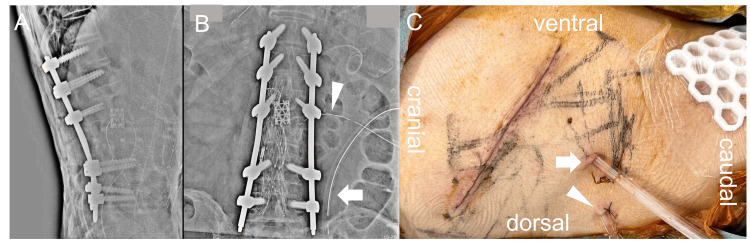
Postoperative lumbar spine radiographs and a photograph of the surgical site (A) lateral view, (B) anterior-posterior view; postoperative lumbar spine radiographs. (C) postoperative photograph of the surgical site. Arrow: 24 Fr dual-lumen tube going to the iliopsoas abscess; arrowhead: 16 Fr dual-lumen tube going to the intervertebral disc.

Based on these findings, the diagnosis was PVO with a right iliopsoas and epidural abscesses. Intravenous meropenem hydrate was empirically started with reference to past blood culture results, and a two-stage surgical approach was planned. The first stage included anterior debridement, anterior fixation using a mesh cage with an autogenous bone graft, and a retroperitoneal CLAP procedure. Anterior fixation was chosen because a large bony defect could be created if the vertebral body with gas-forming infection was sufficiently debrided. Considering that infection control of the L3 vertebral body with gas-forming infection and large iliopsoas abscesses with anterior debridement and fusion may be difficult, we selected to add a retroperitoneal CLAP procedure. The second stage included posterior fixation with a percutaneous pedicle screw technique from the T12 to L5 vertebral body (Figure 3).

**Figure 4:**
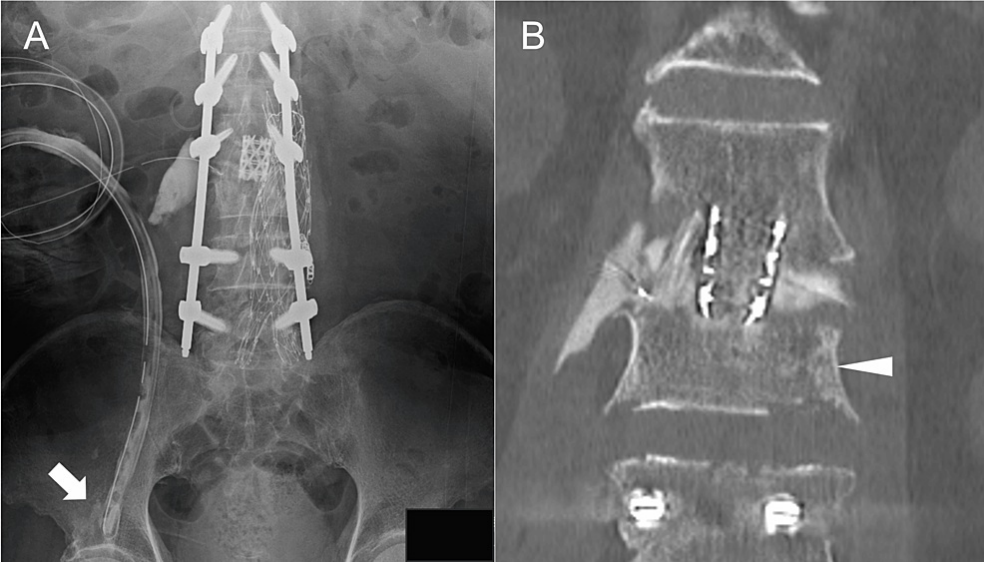
Postoperative contrast test (A) anterior-posterior radiograph, (B) computed tomography A contrast test showing the enhanced small cavity of the iliopsoas abscess (arrow) and the L2-3 intervertebral disc space. Arrowhead: L3 vertebral body

**Figure 5:**
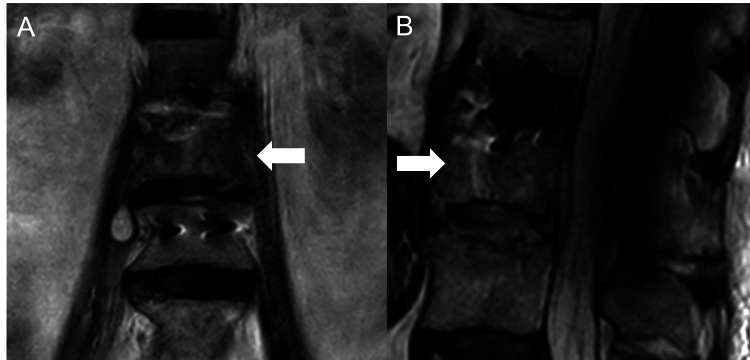
Postoperative magnetic resonance imaging of the lumbar spine (A) coronal image, (B) sagittal image T2-weighted magnetic resonance imaging showed a reduction in the iliopsoas and epidural abscesses. Arrows: L3 vertebral body

The retroperitoneal CLAP procedure was performed with the patient in a left lateral decubitus position to access the right iliopsoas muscle. After an incision along the tenth rib and its resection, the lateral lumbar interbody fusion (LLIF) technique was used to access the psoas major muscle and the L2-3 intervertebral disc. After debridement of the iliopsoas abscess, intervertebral disc, and necrotic vertebral body, a mesh cage filled with autologous iliac bone and ribs was inserted. Subsequently, two dual-lumen tubes (Salem Sump Tube, Cardinal Heath, Dublin, Ohio, US) were inserted percutaneously through a site separate from the original skin incision (Figure 3C). A 16 Fr dual-lumen tube was placed along the retroperitoneal curvature to the intervertebral disc, and its tip was inserted into a pocket created by ligating the scar tissue and the iliopsoas muscle. A 24 Fr dual-lumen tube was inserted into the capsule of the iliopsoas muscle abscess. The wound was closed and covered with negative-pressure wound therapy (NPWT, Renasys; Smith & Nephew Medical, Kingston upon Hull, UK). The suction pressure of the NPWT was set at -40 mmHg. The suction ports of the dual-lumen tubes were connected with a Renasys Y-connector to apply a common negative pressure. Gentamicin (60 mg/50 mL) was continuously administered using a syringe pump at a low-flow rate (2 mL/h) through the dual-lumen tubes [19-21]. Posterior fixation was performed two days after the anterior surgery. Immediately after the surgery, the patient’s lower back pain improved markedly. Rehabilitation was started without restrictions wearing a rigid brace, even during CLAP treatment. One week postoperatively, a contrast test using a diluted nonionic contrast agent (iopamidol) through the two dual-lumen tubes displayed adequate contrast in the iliopsoas abscess cavity and L2-3 intervertebral disc (Figure 4). Iopamidol was selected because it has been reported that it can be administered into the retroperitoneal space. [22] Two weeks postoperatively, CRP laboratory values improved to 0.35 mg/L (Table 1), and CLAP treatment was concluded. He was able to walk with a walker. Blood gentamicin levels were maintained in a safe range during the CLAP treatment (Table 1). Three weeks postoperatively, MRI indicated a substantial reduction of the iliopsoas and epidural abscesses (Figure 5). Six weeks postoperatively, CRP values improved to 0.12 mg/L (Table 1), and antibiotics were changed from intravenous meropenem hydrate (0.5 g every 12 hours) to oral sulfamethoxazole-trimethoprim (2 tablets of 400 mg of sulfamethoxazole and 80 mg of trimethoprim every 12 hours). He was able to walk with a T-shaped cane. Twelve weeks postoperatively, he achieved independent gait.

## Discussion

The present case illustrates two important findings. First, it elucidates that PVO with iliopsoas and epidural abscesses could be treated by combining anterior-posterior fixation with retroperitoneal CLAP. Second, it demonstrates the feasibility of the CLAP procedure for the retroperitoneal space, vertebral body, and intervertebral disc through an approach similar to the LLIF technique.

A previous report demonstrated the feasibility and usefulness of CLAP from the posterior side for surgical site infection in four patients after spinal instrumentation surgery [20]. However, there have been no reports of CLAP from the anterior or lateral side for the approaching vertebral bodies, intervertebral discs, or iliopsoas muscle. For retroperitoneal CLAP, it is necessary to place the tube close to the site of infection to increase the local antimicrobial concentration because the retroperitoneal space is large [23]. In addition, there is a possibility that the tube may deviate from its intended position. In the present case, the placement of the tube in the scar or the iliopsoas muscle prevented tube deviation. The contrast agent injected through the dual-lumen tubes had a contrast effect within the iliopsoas muscle and intervertebral space, confirming that the antibiotics were correctly delivered to the infected site even when placed in the retroperitoneal space, which is sparser and more complex than posterior structures [23]. Moreover, we avoided complications due to the spread of Gentamycin over a large space, which did not occur. If infection control is anticipated, CLAP to other musculoskeletal sites is usually completed within two weeks. Thus, in this case, multiple blood tests were performed within two weeks postoperatively and contrast studies were performed one week postoperatively. However, the optimal timing and frequency of blood tests and contrast studies remain unclear. Further cases are needed to establish the appropriate timing and frequency of these tests to prevent inappropriate perfusion of gentamicin.

Our second key finding is that treatment combining anterior-posterior fixation with retroperitoneal CLAP was effective for PVO with iliopsoas and epidural abscesses. Conventional surgical treatments can sometimes result in poor outcomes [2]. Factors like advanced age, diabetes mellitus, and immunocompromised conditions are associated with severe symptoms of PVO [2]. Furthermore, incomplete debridement, antimicrobial-resistant bacteria, and negative cultures contribute to poorer outcomes in the surgical treatment of PVO [24]. The high concentration of Gentamicin used in CLAP is effective regardless of the susceptibility of bacteria. Therefore, CLAP might be a viable option in cases of suspected resistant bacterial involvement or when choosing the appropriate antibiotic is challenging due to negative cultures. However, spinal fusion surgery, including anterior debridement, is a standard and useful treatment for refractory PVO with severe vertebral body destruction and instability [25,26]. In this case, these treatments were the primary contributors to infection control. Further studies are needed to evaluate the effectiveness and safety of retroperitoneal CLAP.

## Conclusions

This case demonstrates that PVO with iliopsoas and epidural abscesses could be treated by combining anterior-posterior fixation with retroperitoneal CLAP. Additionally, it highlights the clinical feasibility of using retroperitoneal CLAP, or “anterior CLAP in spine surgery.” Retroperitoneal CLAP may be a treatment option for refractory PVO.
